# Distribution of circulating T follicular helper cell subsets is altered in immunoglobulin A vasculitis in children

**DOI:** 10.1371/journal.pone.0189133

**Published:** 2017-12-13

**Authors:** Deying Liu, Jinxiang Liu, Jinghua Wang, Lishuang Guo, Congcong Liu, Yanfang Jiang, Haifeng Wang, Sirui Yang

**Affiliations:** 1 Department of Pediatric Rheumatology and Allergy, The First Hospital, Jilin University, Changchun, China; 2 Department of Rheumatology and Immunology, Wuhan Children’s hospital, Tongji Medical college, Huazhong University of Science & Technology, Wuhan, China; 3 Key Laboratory of Zoonoses Research, Ministry of Education, The First Hospital, Jilin University, Changchun, China; 4 Jiangsu Co-innovation Center for Prevention and Control of Important Animal Infectious Diseases and Zoonoses, Yangzhou, China; Institut Cochin, FRANCE

## Abstract

**Background:**

Immunoglobulin A vasculitis (IgAV) is the most common vasculitis in children. Previously, we demonstrated that patients with IgAV show abnormal proliferation of cluster of differentiation (CD)4^+^C-X-C chemokine receptor type (CXCR)5^+^ follicular helper T (Tfh) cells. Here, we explored the status of Tfh cell subsets and plasma cytokine levels in patients with IgAV.

**Methods:**

CD4^+^CXCR5^+^CD45RA^−^, CD45RA^-^CXCR3^+^CCR6^−^, CD45RA^−^CXCR3^−^CCR6^−^, CD45RA^−^CXCR3^−^CCR6^+^, and CD45RA^−^CXCR3^+^CCR6^+^ Tfh cell fractions and plasma concentrations of interferon-γ, interleukin (IL)-4, and IL-17A were evaluated by flow cytometry and a flow cytometric bead array, respectively, in 30 patients with IgAV and 15 healthy controls (HCs).

**Results:**

Tfh2 and Tfh17 cell fractions were larger and the Tfh2+Tfh17/Tfh1 ratio and plasmaIL-4 and -17A levels were higher in patients with IgAV than in the HCs. Only Tfh1 cell counts were reduced in the abdominal subtype. The elevations in Tfh2 and Tfh17 cell counts and plasma IL-4 levels were abrogated by treatment. Tfh2 cell number was positively correlated with serum IgA and plasma IL-4 levels, but negatively correlated with the serum C4 concentration, while Tfh17 cell number was positively correlated with the serum IgA level and Tfh2 cell counts.

**Conclusions:**

Abnormally high numbers of Tfh2 and Tfh17 cells are linked to the occurrence and development of IgAV, but are not specific to the abdominal type. Only Tfh1 cells play a critical role in abdominal-type IgAV.

## Introduction

Immunoglobulin A vasculitis (IgAV), formerly known as Henoch–Schönlein purpura, is an IgA-associated small-sized vessel leukocytoclastic vasculitis (LCV) with non-thrombocytopenic palpable purpura (mainly involving the lower extremities, although lesions are not restricted to this area). IgAV is the most common form of vasculitis in children, with an estimated annual incidence of 30–267 cases per 100,000 children [1,2]. The disease can be triggered by chlamydia, bacteria, viruses, mycoplasma, or parasitic agents infection. Clinical manifestations predominantly involve the skin, joints, gastrointestinal tract, and kidneys, and occasionally other organs, and can be severe [2,3]. Progressive impairment of renal function, bowel perforation, and central nerve system involvement are rare, but constitute the major causes of IgAV-associated morbidity. Many patients experience abdominal pain as an initial symptom, which can complicate clinical diagnosis. Therapy for IgAV is mostly supportive and symptomatic, because the disease is usually benign and self-limiting. However, a subset of cases have a remitting–relapsing course, especially those with recurrent abdominal pain or steroid reduction difficulties; aggressive therapies such as glucocorticoids and/or immunosuppressants are indicated under these conditions [1]. Aberrant deposition of glycosylated IgA_1_ and complement activation in the walls of small vessels, together with subsequent activation of an alternate complement pathway, play an important role in the etiology of IgAV [4–6]. Several studies have demonstrated that hyperactivation of Th2 and Th17 cells, as well as a decline in autoreactive natural killer cell number, may also be contributing factors [7], because these cells are key players in the humoral immune response. Additionally, increased serum interleukin (IL)-4, -6, and -17 concentrations have also been found in patients with IgAV [8,9].

Follicular helper T (Tfh) cells are critical for the formation of germinal centers (GCs), immunoglobulin (Ig) class-switch recombination, somatic hypermutation, and differentiation of B cells into long-lived memory B cells and plasma cells [10–12]. Tfh cells can be distinguished from other cluster of differentiation (CD)4^+^ T cell lineages by their high expression of chemokine receptor C-X-C chemokine receptor type (CXCR)5, programmed death-1, inducible costimulator (ICOS), signaling lymphocytic activation molecule adapter protein, B and T lymphocyte attenuator, CD40 ligand, and IL-21 [13–15]. Tfh cells act coordinately with B cells, and dysregulation of their interaction can result in autoimmunity or immunodeficiency. Circulating Tfh cells have been implicated in various autoimmune diseases [16,17]. Our and other research groups have previously reported aberrant expansion of CD4^+^CXCR5^+^ Tfh cells in patients with IgAV [18,19]; strategies that reduce Tfh cell generation improve symptoms in some autoimmune disease models [15]. Determining the characteristics of different Tfh cell subsets in patients with IgAV is important for the development of more effective treatments.

We identified a population of CD3^+^CD4^+^CXCR5^+^ Tfh cells within the memory T cell compartment (CD45RA^−^) of human peripheral blood [20,21]. Four subsets of CD4^+^CXCR5^+^CD45RA^−^ Tfh cells have been phenotyped according to their C-C chemokine receptor (CCR)6 and CXCR3 expression: CD45RA^−^CXCR3^+^CCR6^−^ (Tfh1), CD45RA^−^CXCR3^−^CCR6^−^ (Tfh2), CD45RA^−^CXCR3^−^CCR6^+^ (Tfh17), and CD45RA^−^CXCR3^+^CCR6^+^ (Tfh1/17). Of these, only Tfh2 and Tfh17 cells can induce immunoglobulin production by B cells via IL-21 [15,22].

In the present study, we investigated the distribution of distinct Tfh cell subsets in skin, abdominal, and kidney-type IgAV and examined the correlation between the different subsets and clinical parameters. We also evaluated plasma interferon (IFN)-γ and IL-4 and -17A levels and their association with Tfh cell subsets.

## Materials and methods

### Patients

Written informed consents were obtained from parents or guardians of study participants. The experimental protocol followed the guidelines of the Declaration of Helsinki and was approved by the Human Ethics Committee of Jilin University (Jilin University, Changchun, China). A total of 30 patients with active IgAV were recruited at the inpatient service of the Department of Pediatrics, the First Hospital of Jilin University from October 2015 to July 2016, and met the following criteria: (1) children younger than 18 years old; (2) met the European League Against Rheumatism/Pediatric Rheumatology International Trials Organization/Pediatric Rheumatology European Society criteria for IgAV [23]; i.e., palpable purpura (mandatory) and one of following findings: histopathology (typical LCV with predominant IgA deposits or proliferative glomerulonephritis with predominant IgA deposits); diffuse abdominal pain (abdominal involvement); acute arthritis or arthralgia (joint involvement); proteinuria > 0.3 g/24 h or > 30 mmol/mg of urine albumin/creatinine ratio on a spot morning sample (renal involvement); hematuria [> 5 red blood cells (RBCs)/high-powered field or ≥ 2+ on dipstick or RBC casts in urinary sediment]; mild nephropathy; i.e., microhematuria (≥ 5 RBCs/high-powered field) and/or proteinuria that did not reach the nephrotic range; severe nephropathy, defined as a) nephrotic syndrome or b) nephritic syndrome; and renal insufficiency (plasma creatinine > 125% of the upper limit of normal). On the basis of the presenting symptoms, the 30 patients in this study were divided into skin (n = 9), abdominal (n = 12), kidney (n = 4), joint (n = 3), and mixed (patients presenting two or more non-purpura symptoms; n = 2) IgAV types.

Given the self-limiting and benign course of IgAV, symptom-oriented and supportive therapies were administered to patients following admission. Glucocorticoids and/or immunosuppressants were administered to those presenting severe gastrointestinal complications or proliferative glomerulonephritis. Remission following treatment was defined according to two criteria: (1) after 2 weeks, all skin purpura improved, with no appearance of new rashes; and (2) abdominal pain, intestinal wall edema, arthralgia, hematuria, and/or proteinuria and other related symptoms were alleviated. Only 27 of the total patients entered remission. There were three patients with recurrent skin purpura or abdominal pain. We randomly selected 15 patients at the remission stage. The prognosis of IgAV is mostly benign; therefore, blood samples were collected from patients who had successfully entered remission. The 15 patients in remission phase were of skin (n = 4), abdominal (n = 7), kidney (n = 2), joint (n = 1), and mixed (n = 1) types.

A total of 15 sex- and age-matched healthy controls (HCs) were recruited for the study. All participants underwent a routine blood test: measurement of serum immunoglobulin and complement levels using a specific protein analyzer (BN-II; Siemens, München, Germany), serum C-reactive protein (CRP) level using the QuikRead go CRP kit (Orion Diagnostica, Espoo, Finland), urinary protein level using a P800 biochemical analyzer (Roche, Mannheim, Germany), and urinary RBC and white blood cell (WBC) counts using a UF-1000 automatic urinary sediment analyzer (Sysmex, Kobe, Japan).

### Cell isolation

Fresh venous blood samples were collected from HCs and patients with IgAV following treatment, as well as from patients in remission. Peripheral blood mononuclear cells (PBMCs) were isolated from subjects by density-gradient centrifugation using Ficoll-Paque Plus (Amersham Biosciences, Little Chalfont, UK) at 800 × *g* for 30 min at 25°C.

### Flow cytometry

PBMCs at 4 × 10^6^/ml were analyzed by multicolor flow cytometry (FACSAria II; BD Biosciences, Franklin Lakes, NJ, USA). Human PBMCs (10^6^/tube) were stained with BV510-anti-CD3 (clone: UCHT1), allophycocyanin (APC)-H7-anti-CD4 (clone: RPA-T4), BB515-anti-CXCR5 (clone: RF8B2), phycoerythrin (PE)-Cy5-anti-CD45RA (clone: HI100), PE-Cy7-anti-CCR6 (clone: 11A9), or APC-anti-CD183 (clone: IC6/CXCR3) (Becton Dickinson, San Jose, CA, USA) at room temperature for 30 min. Data were processed using FlowJo v.5.7.2 software (Tree Star, Ashland, OR, USA).

### Cytometric bead array (CBA) analysis of plasma cytokines

Plasma IFN-γ and IL-4 and -17A concentrations were determined using the CBA Human Soluble Protein Master Buffer kit (BD Biosciences) on a flow cytometer. A standard curve was generated for each set of reagents. The minimum and maximum detection limits for all six cytokines were 1.0 and 10,000 pg/ml, respectively. Quantification was performed using CellQuest Pro and CBA software (Becton Dickinson).

### Statistical analysis

Data are expressed as the median and range. Differences among groups were evaluated by one-way analysis of variance. The Student’s unpaired or paired t test was performed to compare two groups, and the Mann-Whitney U test was performed for nonparametric data. Relationships between variables were analyzed by Spearman’s rank correlation test. Statistical analyses were performed using SPSS v.19.0 software. Two-sided P values < 0.05 were considered statistically significant.

## Results

### Clinical characteristics

The demographic and clinical characteristics of the study subjects are shown in Table 1. All patients presented with palpable skin purpura, especially on the lower extremities and buttocks. Among the patients, 30.0% presented with skin purpura (skin type), 40.0% with gastrointestinal tract discomfort (abdominal type), 13.33% with microhematuria and/or mild proteinuria (1+ to 2+) (kidney type), 10.0% with arthralgia and/or arthritis (joint type), and 6.67% with two or more non-purpura symptoms (mixed type). Upon recruitment, the WBC count (P < 0.0001), and the platelet (P = 0.0069), serum IgA (P = 0.0173), IgE (P = 0.0402), and complement C4 (P = 0.0451) levels were higher in patients with IgAV than in the HCs (Table 1). No sequelae or other complications were noted. Etiological factors in abdominal-type IgAV were upper respiratory tract infection (URTI) (n = 8), *Helicobacter pylori* infection (n = 3), and allergy (n = 1); etiological factors in the kidney type were URTI (n = 3) and other unknown causes (n = 1) (data not shown).

**Table 1:** Demographic and clinical characteristics of the study subjects.

	IgAV (n = 30)	Healthy controls (n = 15)
Age, year	6 (3–14)	6 (3–13)
Female/male	16/14	7/8
WBC, 10^9^/l	9.82 (3.97–18.23)*	7.1 (5.13–9.11)
Lymphocytes, 10^6^/l	3.36 (1.6–5.43)	3.61 (1.36–4.28)
Platelet, g/l	367 (179–471)*	278 (167–389)
Serum IgA, g/l	2.25 (0.97–5.83)*	1.56 (0.95–3.89)
Serum IgG, g/l	10.4 (0.98–17.1)	10.1 (1.15–16.02)
Serum IgM, g/l	1.23 (0.67–3.12)	1.02 (0.69–3.43)
Serum IgE, g/l	51.2 (23.9–637.0)*	32.1 (19.3–80.4)
Serum C3, g/l	1.31 (0.82–1.73)	1.42 (0.95–1.61)
Serum C4, g/l	0.41 (0.19–0.47)*	0.33 (0.15–0.51)
Serum CRP (mg/l)	7.53 (1.28–70.12)*	3.1 (0.82–4.2)

CRP, C-reactive protein; WBC, white blood cell.

*P < 0.05 *vs*. healthy controls (values before treatments)

### Detection of circulating Tfh cell subsets in IgAV patients

**Fig 1:**
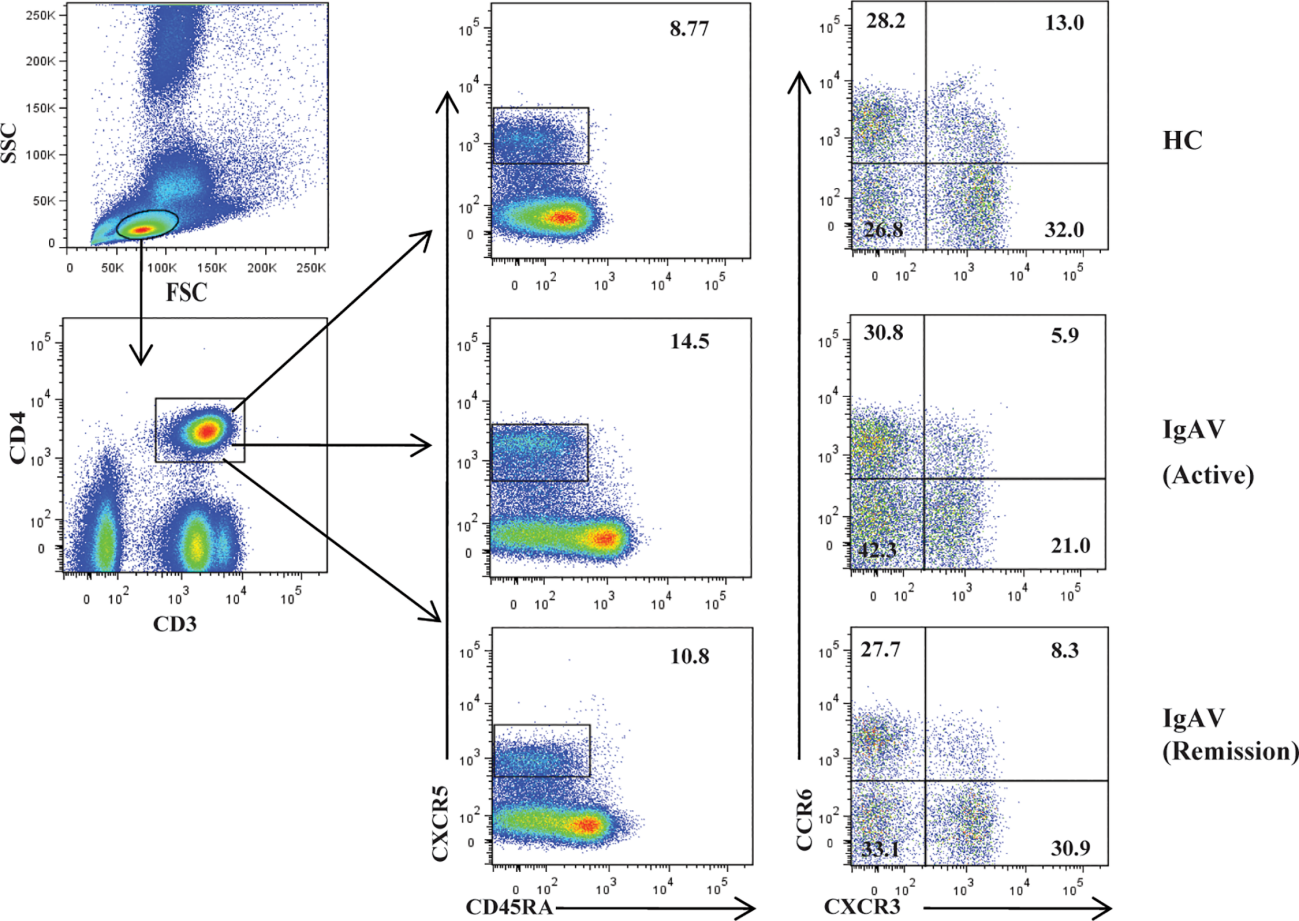
Detection of circulating Tfh cell subsets by flow cytometry. Peripheral blood mononuclear cells (PBMCs) were isolated from patients with immunoglobulin A vasculitis (IgAV) (n = 30) and age and gender-matched healthy controls (HCs; n = 15), labeled with fluorophore-conjugated antibodies, and analyzed by flow cytometry. A gating strategy was used to identify CD3^+^CD4^+^ T helper (Th) and CD4^+^CXCR5^+^CD45RA^−^, CD45RA^−^CXCR3^+^CCR6^−^, CD45RA^−^CXCR3^−^CCR6^−^, CD45RA^−^CXCR3^−^CCR6^+^, and CD45RA^−^CXCR3^+^CCR6^+^ follicular helper T (Tfh) cell subsets.

**Fig 2:**
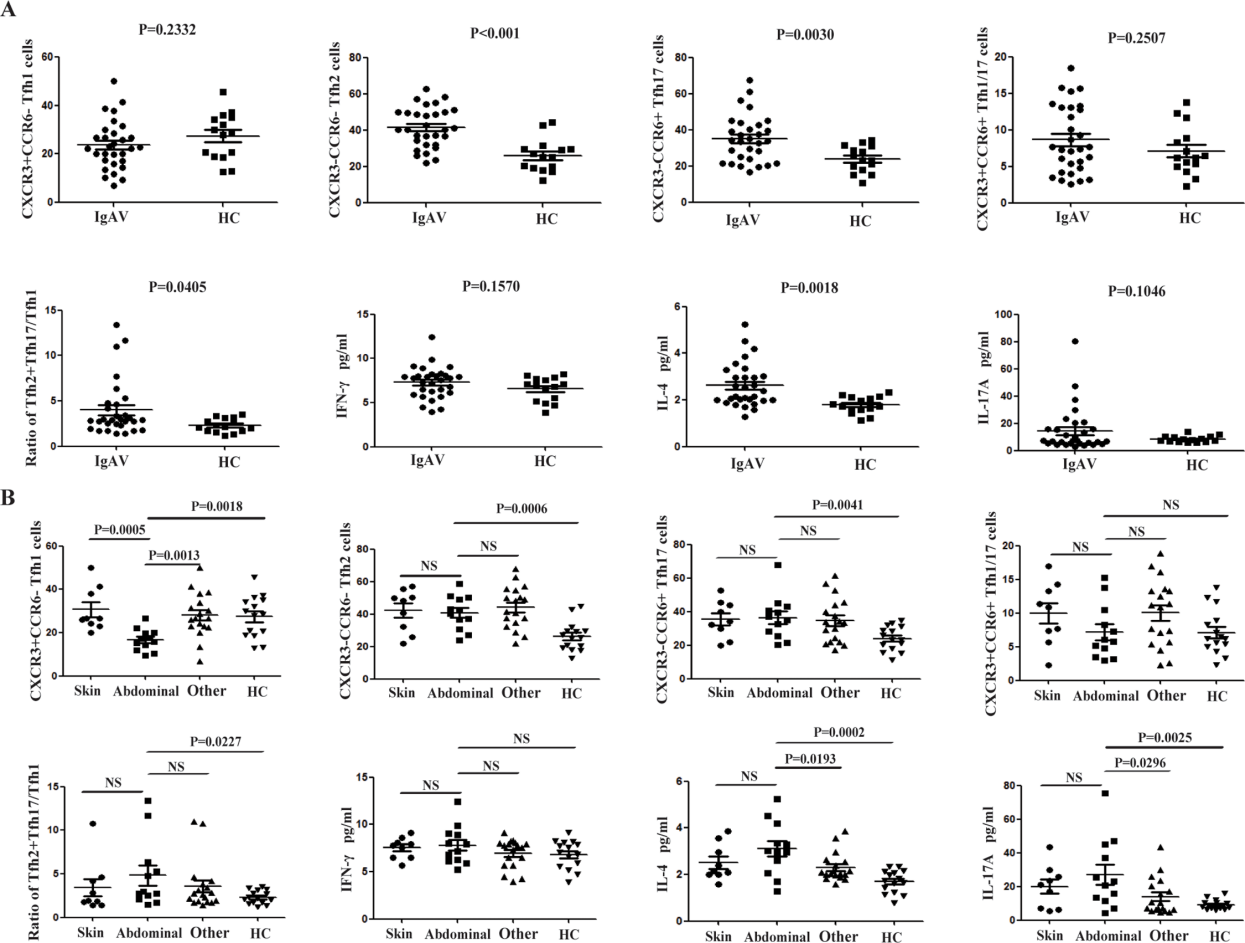
Association between follicular helper T (Tfh) cell subsets and immunoglobulin A vasculitis (IgAV) symptoms and treatment strategies. (A) Tfh cell subsets and plasma IFN-γ and IL-4 and -17A levels were compared in IgAV patients and healthy controls (HCs). (B) Tfh cell subset and cytokine detection in skin, abdominal, other (non-abdominal) IgAV types and HC. *P < 0.05, **P < 0.01; NS, not significant.

To investigate the status of circulating Tfh cells in IgAV, we detected the levels of four subsets of memory CD4^+^CXCR5^+^CD45RA^−^ Tfh cells, including CD45RA^−^CXCR3^+^CCR6^−^ Tfh1, CD45RA^−^CXCR3^−^CCR6^−^ Tfh2, CD45RA^−^CXCR3^−^CCR6^+^ Tfh17, and CD45RA^−^CXCR3^+^CCR6^+^ Tfh1/17 cells, which were gated from CD3^+^CD4^+^ Th cells in a flow cytometry analysis of 30 patients with IgAV and HCs(Fig 1).Circulating Tfh2 and Tfh17 cells counts and Tfh2+Tfh17/Tfh1 ratios were increased in IgAV relative compared with those in the HCs (P < 0.001, P = 0.0030, and P = 0.0405, respectively) (Fig 2A); however, the number of circulating Tfh1/17 cells did not differ between the two groups (P > 0.05; Fig 2A). We also found that Tfh1 cell counts were decreased only in the abdominal-type (P = 0.0018; Fig 2B) of IgAV relative to that in the HCs. Meanwhile, there were significant differences in Tfh1 cell counts between abdominal-type and skin and other (non-abdominal-type) IgAV (P = 0.0005 and 0.0013, respectively) (Table 2).

**Table 2:** Tfh cell counts and plasma cytokine levels in IgAV and healthy control groups [median ± standard deviation (range)].

	IgAV (n = 27)	Healthy controls (n = 15)
Tfh1 cell, 10^6^/l	23.08± 10.19 (6.83–50.08)	28.45± 9.63 (12.78–45.6)
Tfh2 cell, 10^6^/l*	42.81± 11.89 (21.87–67.73)	25.27± 8.96 (12.57–44.59)
Tfh17 cell, 10^6^/l*	33.87± 13.48 (17.0–67.82)	23.17± 7.21 (11.03–34.47)
Tfh1/17 cell, 10^6^/l	6.15± 3.31 (2.25–13.73)	7.68± 4.52 (2.59–18.45)
Plasma IFN-γ, pg/ml	7.75± 1.82 (3.97–12.46)	7.02± 1.38 (3.84–8.22)
Plasma IL-4, pg/ml*	2.15± 0.97 (1.29–5.23)	1.73± 0.36(1.12–2.34)
Plasma IL-17A, pg/ml	7.92± 15.79 (2.73–80.57)	7.71± 2.32(5.9–13.67)

*P < 0.05 *vs*. healthy controls (values before treatments)

### Association between plasma cytokine levels and IgAV symptoms

Plasma levels of IL-4 (P = 0.0018; Fig 2A) but not IFN-γ or IL-17A (P > 0.05; Fig 2A) were elevated in the IgAV and HC groups (Table 2). Compared with the levels in the HCs, the IL-17A concentration was increased in abdominal-type IgAV (P = 0.0025), which differed from that in the other (non-abdominal) types of IgAV (P = 0.0296); however, this effect was non-specific, as it was also detected in the skin type (P > 0.05; Fig 2B).

### Alterations in Tfh subsets and plasma cytokine levels following treatment

**Fig 3:**
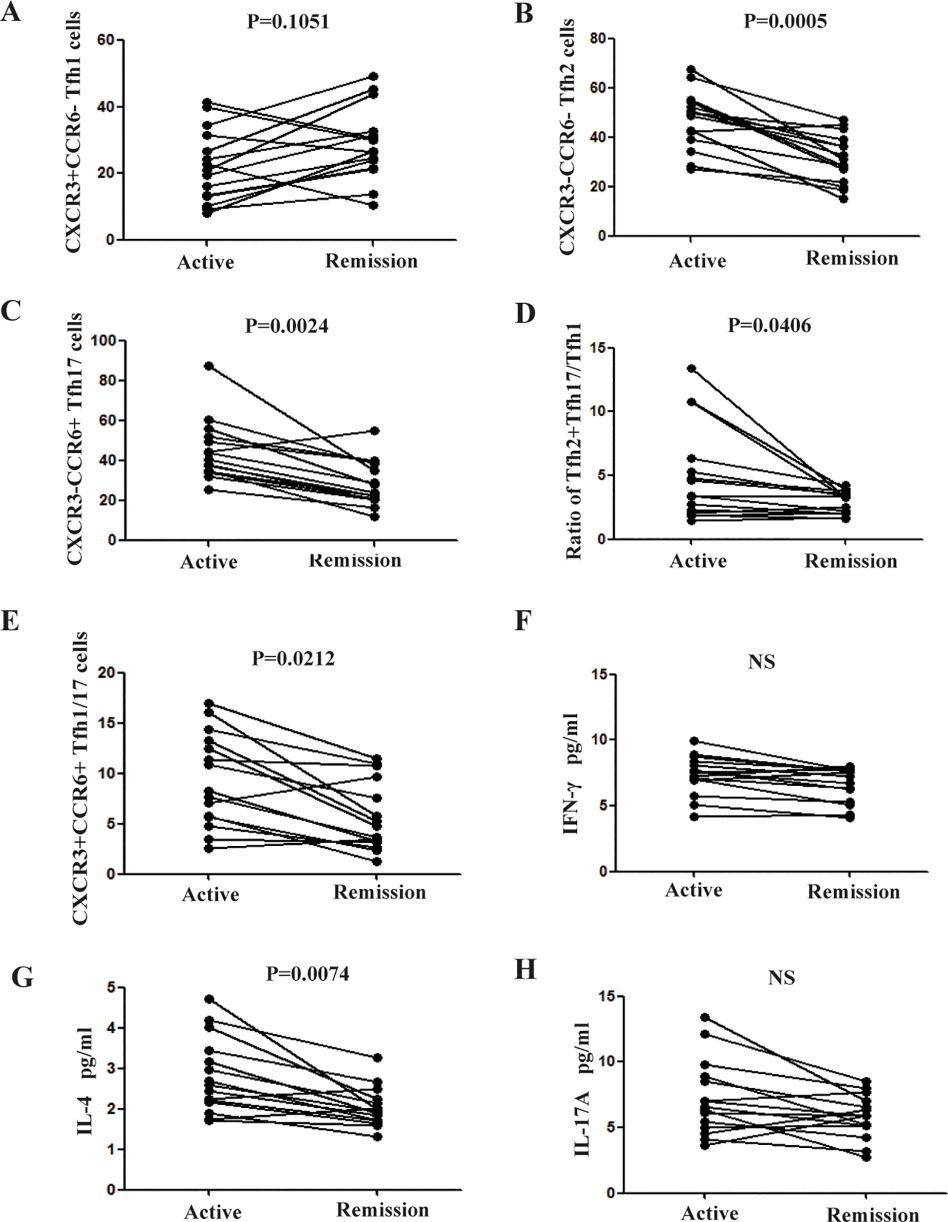
Treatment-induced changes in follicular helper T (Tfh) cell subsets and plasma cytokine levels. Following treatment, 15 patients showed disease remission. Tfh cell counts and plasma IFN-γ and IL-4 and -17A levels were compared between active and remission stages.

Following symptom-oriented and supportive therapies, the majority of patients showed improvement. We examined Tfh cell subsets in 15 patients in remission (Fig 3). Tfh2 and Tfh17 counts, plasma IL-4 levels, and the Tfh2+Tfh17/Tfh1 ratio were reduced relative to the absolute value in the active stage (P = 0.0005, 0.0024, 0.0074, and 0.0406, respectively). Although the number of Tfh1 cells (P > 0.05; Fig 3A) did not change during remission, 8/15 patients in the remission stage were of abdominal-type IgAV, and Tfh1 cell counts were increased in these abdominal-type IgAV patients during remission compared with that in the active stage (P = 0.0437, data not shown). There were no changes in IFN-γ (P > 0.05; Fig 3F) and IL-17A (P > 0.05; Fig 3H) levels during remission. Interestingly, although the numbers of Tfh1/17 cells were similar between patients with IgAV and the HCs, they were reduced by treatment (P = 0.0212; Fig 3E).

### Association between and among Tfh cell subsets and clinical parameters

**Fig 4:**
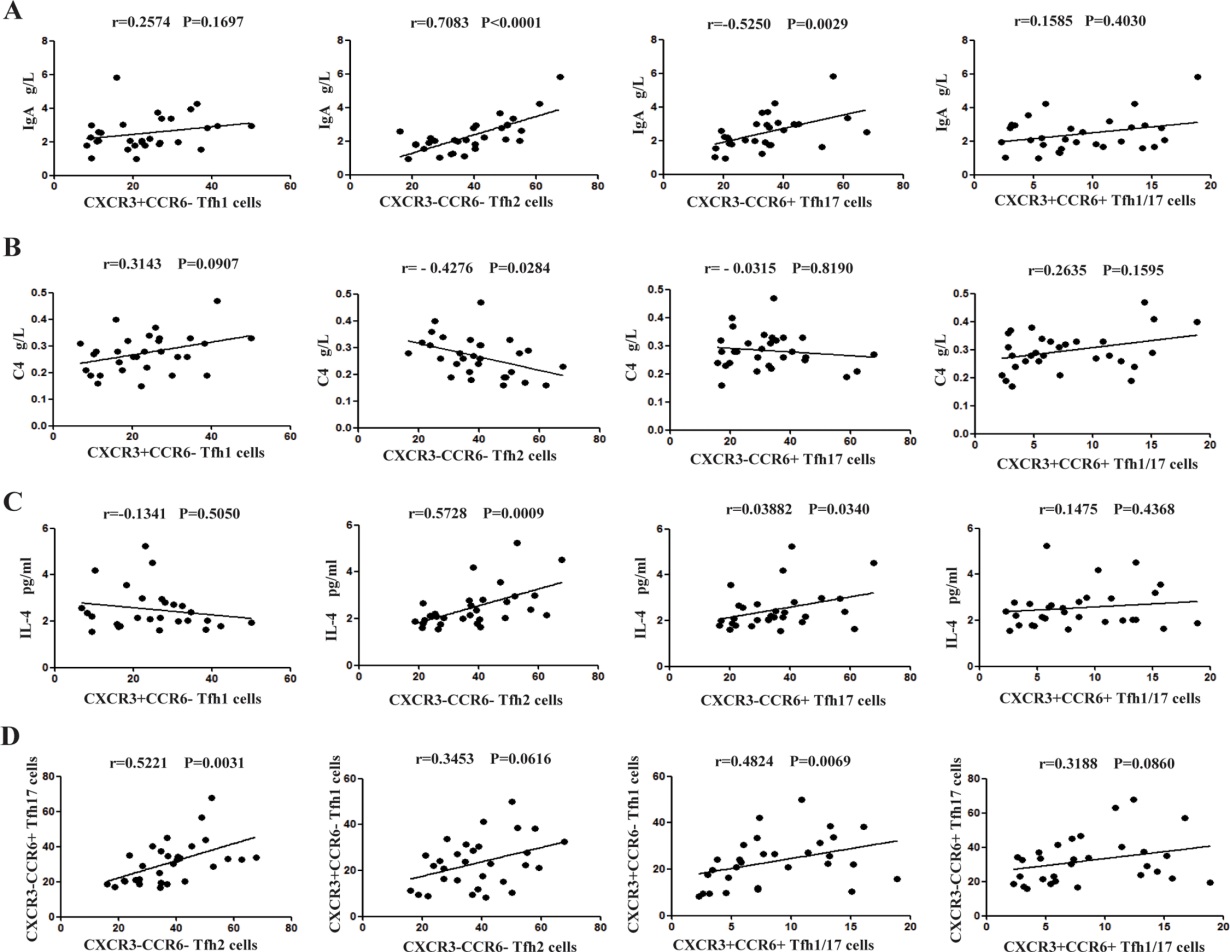
Correlation between and among follicular helper T (Tfh) cell subsets and serum IgA or plasma IL-4 levels. Correlations between indicated Tfh cell subsets and (A) serum IgA, (B) plasma C4, (B) IL-4, and (D) among Tfh cell subsets were analyzed by Spearman’s rank correlation test.

We investigated whether alterations in Tfh cell subsets were associated with disease etiology and progression, and found that the number of Tfh2 (r = 0.7083, P < 0.001) and Tfh17 (r = 0.5250, P = 0.0029), but not of Tfh1 (r = 0.2574, P = 0.1697) or Tfh1/17 (r = 0.1585, P = 0.4030), cells was positively correlated with serum IgA levels (Fig 4A). Circulating Tfh2 cell counts were also positively correlated with plasma IL-4 levels (r = 0.5728, P = 0.0009; Fig 4C) and Tfh17 cell counts (r = 0.5221, P = 0.0031; Fig 4D). By contrast, circulating Tfh2 cell counts were inversely related to serum C4 levels (r = −0.4276, P = 0.0284; Fig 4B), but not the number of Tfh1 cells (r = 0.3453, P = 0.0616; Fig 4D). We also explored the relationship between Tfh cell subsets and serum IgA level/C4 in the HCs (data not shown).There was no correlation between Tfh 2 (r = 0.2006, P = 0.4736) and Tfh17 (r = 0.3091, P = 0.2623) cell subsets with serum IgA level in the HCs. Similarly, Tfh 2 (r = -0.1587, P = 0.5720) and Tfh17 (r = 0.1589, P = 0.5717) cell subsets showed no association with serum C4 in the HCs, respectively.

## Discussion

IgAV is a common childhood vasculitis triggered by environmental and genetic factors, and is associated with a history of URTI [3]. It has long been presumed that aberrant deposition of glycosylated IgA_1_ and complement activation contribute to IgAV. Recently, Tfh cells have been identified as a novel cell type that promotes isotype switching and co-operates with B cells in GCs [24]. These cells mediate adaptive immune responses in various human autoimmune diseases such as rheumatoid arthritis, systemic lupus erythematosus, and multiple sclerosis [9, 17, 25], with different cytokines, transcription factors, and pathogens activating distinct Tfh cell subsets. The expression of chemokine receptors has been instrumental in defining human CD4^+^T cell subsets. The expression of CXCR3 is preferentially maintained by cells committed to the Th1 pathway, whereas CCR6 is expressed by Tfh17 cells. In CD4^+^CXCR5^+^ T cells, CXCR3^+^CCR6^-^ cells expressed T-bet, a transcription factor of Th1 cells; CXCR3^−^CCR6^−^ cells expressed GATA3, a transcription factor of Th2 cells; whereas CXCR3^−^CCR6^+^ cells expressed RORγT, a transcription factor of Th17 cells[26,27]. An altered balance of circulating Tfh cell subsets has been linked to IgAV. The prognosis of IgAV is mainly dependent on the extent of kidney damage; however, when there is abdominal involvement (without skin purpura) as an initial symptom or recurrent episodes of abdominal pain, a rapid diagnosis is critical. In our previous work, we demonstrated that Tfh cell subpopulations contribute to the development of IgAV in different ways. However, it is unknown whether certain Tfh cell subsets are specific to abdominal and kidney-type IgAV.

The present study focused on the mechanisms underlying abdominal and kidney-type IgAV. Expansion of CD4^+^CXCR5^+^ICOS^+^ Tfh cells has been implicated in IgAV [18,19,28]. However, this was concluded based on the direct detection of total CD3^+^CD4^+^ Th cells or the indirect detection of related cytokines. In the present study, we examined various Tfh cell subsets in IgAV and found that they contributed differentially to IgAV pathogenesis and remission. We also demonstrated that the balance of CD3^+^CD4^+^CD45RA^−^ Tfh cells is altered in patients with gastrointestinal-specific IgAV. Many patients experience abdominal pain as an initial symptom, which can complicate the clinical diagnosis. In addition, the progression of the abdominal symptoms is rapid and with more complications. Therefore, we think it is important to identify the unique pathogenesis of the abdominal type of IgAV.

Th2 cellscontrol immunity to extracellular parasites and all forms of allergic inflammatory response, including IgAV. Unlike total T helper cells, Tfh2 and Tfh17 cells can provide central assistance to naïve B cells via IL-21 production, resulting in the secretion of various Ig isotypes. Interestingly, in the present study, we found that the numbers of Tfh2 and Tfh17 cells in all types of IgAV patients were higher than those in the HCs, and were reduced following treatment during disease remission, thereby shifting the Tfh2+Tfh17/Tfh1 balance towards a proinflammatory response. However, Tfh2 and Tfh17 cell counts did not differ between abdominal and skin/non-abdominal types, nor between kidney and skin/non-kidney types (data not shown), implying that these circulating cells play a key role in IgAV, but not of a specific type. In addition, the numbers of circulating Tfh2 and Tfh17 cells were positively correlated with serum IgA levels, while Tfh2 cell numbers were inversely correlated with serum C4 levels in patients with IgAV. C3 and C4 are also known to be deposited in late vasculitis lesions. Complement activation via alternative and lectin pathways is observed in patients with IgAV nephritis and may initiate the inflammatory cascade, thereby worsening glomerular injury [2, 29].Abnormal elevation of Tfh2 cell numbers may exacerbate C4 depletion, resulting in its deposition on vascular walls, which has been linked to the occurrence of IgAV. In addition, Tfh2 cell and Tfh17 cell numbers were positively correlated. Given the important role of Tfh cells in humoral immunity, a balance between stimulatory and inhibitory mechanisms is required to regulate the function of Tfh cells and maintain immune homeostasis [30]. Taken together, the above findings suggested that Tfh2 and Tfh17 cells are involved in the occurrence and development of IgAV. Despite the diversity of symptoms, the most urgent and easily relapsed cases are of the abdominal type.

Interestingly, we found that Tfh1 cell counts were reduced in the abdominal type compared with those in the other types of IgAV, and increased in the remission stage relative to that in the active stage (data not shown). Tfh1 cells are proinflammatory effectors of the autoimmune response, as they lack the capacity to act coordinately with naïve B cells [30–32]. A decrease in Tfh1 cell numbers may weaken the immune defense reaction. However, migration of Tfh1 cells into inflamed organs as a result of CXCR3 expression might contribute to the reduced number of Tfh1 cells in peripheral blood. Indeed, kidney infiltration by CXCR3-expressing CD4^+^ T cells has also been detected in patients with active lupus nephritis [9], suggesting that the pathogenesis of the abdominal type is closely related to Tfh1 cells; refractory cases in particular merit further exploration. Combined with the discovery of Tfh2 and Tfh17 cells, we also demonstrated the importance of immune dysfunction in the pathogenesis of IgAV. Our results highlight the association between systemic immunity and IgAV pathogenesis.

Excess cytokine production can cause host tissue injury in infective and sterile forms of inflammation [33]. In a proinflammatory environment, Th2 cells can secrete IL-4, -5, and -13 to counteract this event [15, 34, 35]. The Th cell cytokines IFN-γ and IL-17 are linked to the development of autoimmune disease [36]. Increased serum IL-17 levels and peripheral Th17 cell counts have been noted in children with active IgAV [9]. The observed increases in IL-4 and -17A levels in abdominal-type IgAV indicate that these cytokines are involved in disease occurrence and development, but are not necessarily specific to this type. However, plasma IL-17A levels were not increased in the kidney type; this may be a result of the small number of patients of each clinical type that were examined. Under inflammatory conditions, Th17 lymphocytes shift towards a Th1 or Th2 phenotype, acquiring the ability to produce IFN-γ or IL-4, and are more pathogenic than in unshifted cells [37, 38]. Circulating Tfh2 cell counts were positively correlated with plasma IL-4 levels, leading us to speculate that feedback by the IL-4 produced by these cells induces the amplification of the inflammatory response, while a partial increase in the number of Tfh17 cells by IL-4 causes these cells to differentiate into Tfh2 cells. The major determinant of Th cell differentiation is the presence of cytokines at the time of naïve T cell activation. Concurrently, IL-4 also can inhibit Th1 cell differentiation and induce Th2 cells. Interfering with IL-4 signaling in Tfh cells during inflammation is a promising therapeutic strategy to block their differentiation into pathogenic Tfh cells in patients with IgAV. We speculated that pathogen load, immune homeostasis, and disease type affect the composition of the responding Tfh cell population. The heterogeneity of patients’ symptoms indicated that distinct Tfh cells and molecular mechanisms underlie IgAV development.

Our findings suggested that Tfh2 and Tfh17 cells play a crucial role in the pathogenesis of IgAV, but are not specific to the abdominal type. Differential activation of Tfh cell subsets is responsible for the distinct clinical subtypes in IgAV patients, and may account for differences in individual responses to therapies. In particular, the abdominal type might have a unique pathogenesis. In particular, there is no rash in the early stage of IgAV; therefore, the detection of abnormal markers of Tfh cell subsets will aid diagnosis. The primary purpose of therapy is to ease acute symptoms and reduce the risk of recurrence; understanding the characteristics of different Tfh cell subsets in IgAV could allow clinicians to prescribe more individualized therapeutic regimens. A limitation of our study was the small number of patients of the abdominal type; additional investigations with larger cohorts could help to validate our observations and clarify whether different Tfh subsets can serve as useful prognostic markers for IgAV.

## Supporting information

S1 Fig The circulating Tfh cell subsets by flow cytometry of 22 patients in the active stage. (TIF)

S2 Fig The circulating Tfh cell subsets by flow cytometry of the other 8 patients in the active stage and all patients in remission stage. (TIF)

S3 Fig The circulating Tfh cell subsets by flow cytometry of HCs. (TIF)

S4 Fig Plasma IFN-γ and IL-4 and -17A levels in all the IgAV patients and HCs. (TIF)
