# Supplementary figures and images for: Distribution of circulating T follicular helper cell subsets is altered in immunoglobulin A vasculitis in children
Source: PLoS One. 2017 Dec 13;12(12):e0189133. doi: 10.1371/journal.pone.0189133 (PMC5728569; doi:10.1371/journal.pone.0189133)

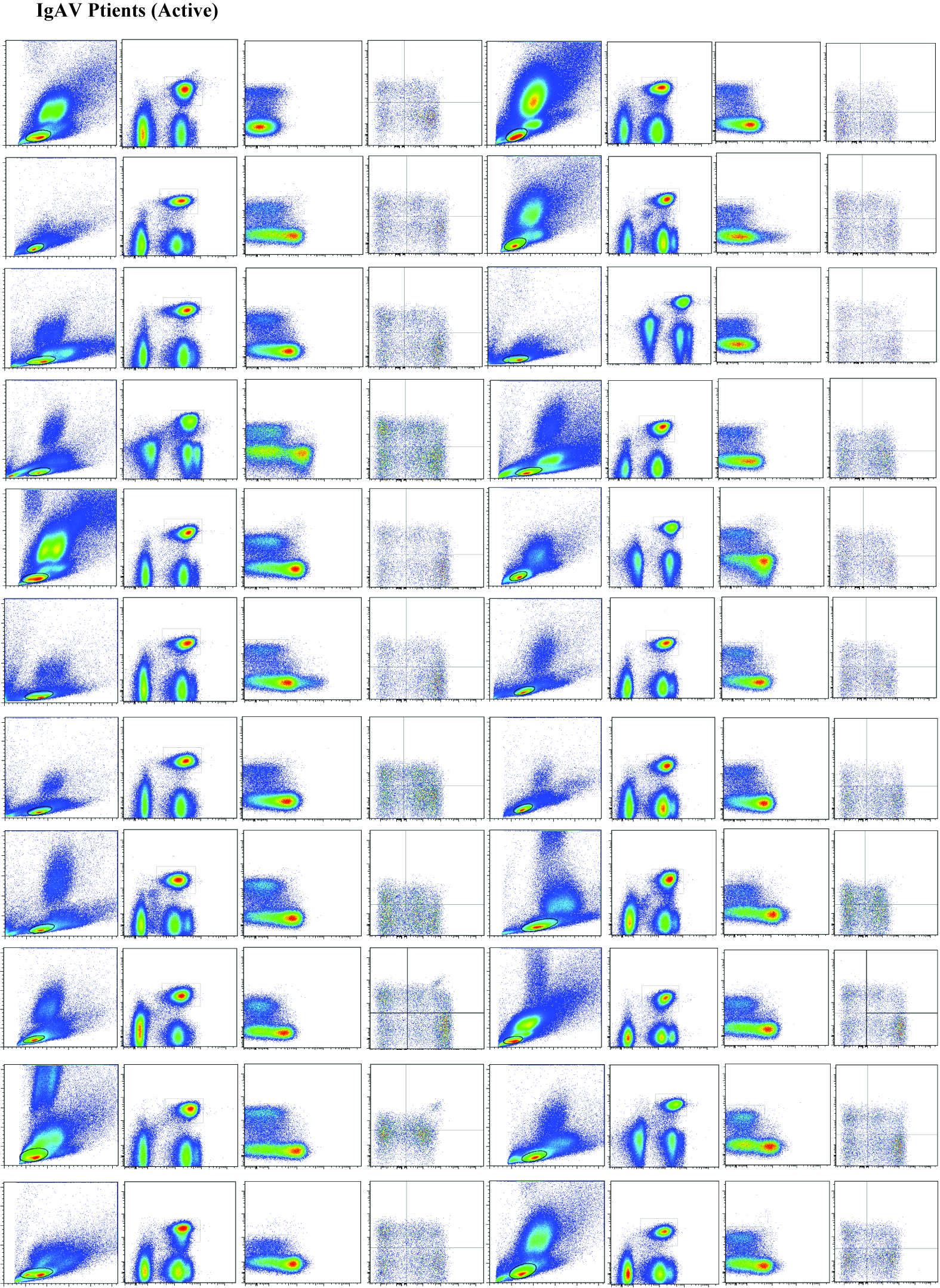

Supplement: S1 Fig — (TIF) [file pone.0189133.s001.tif]

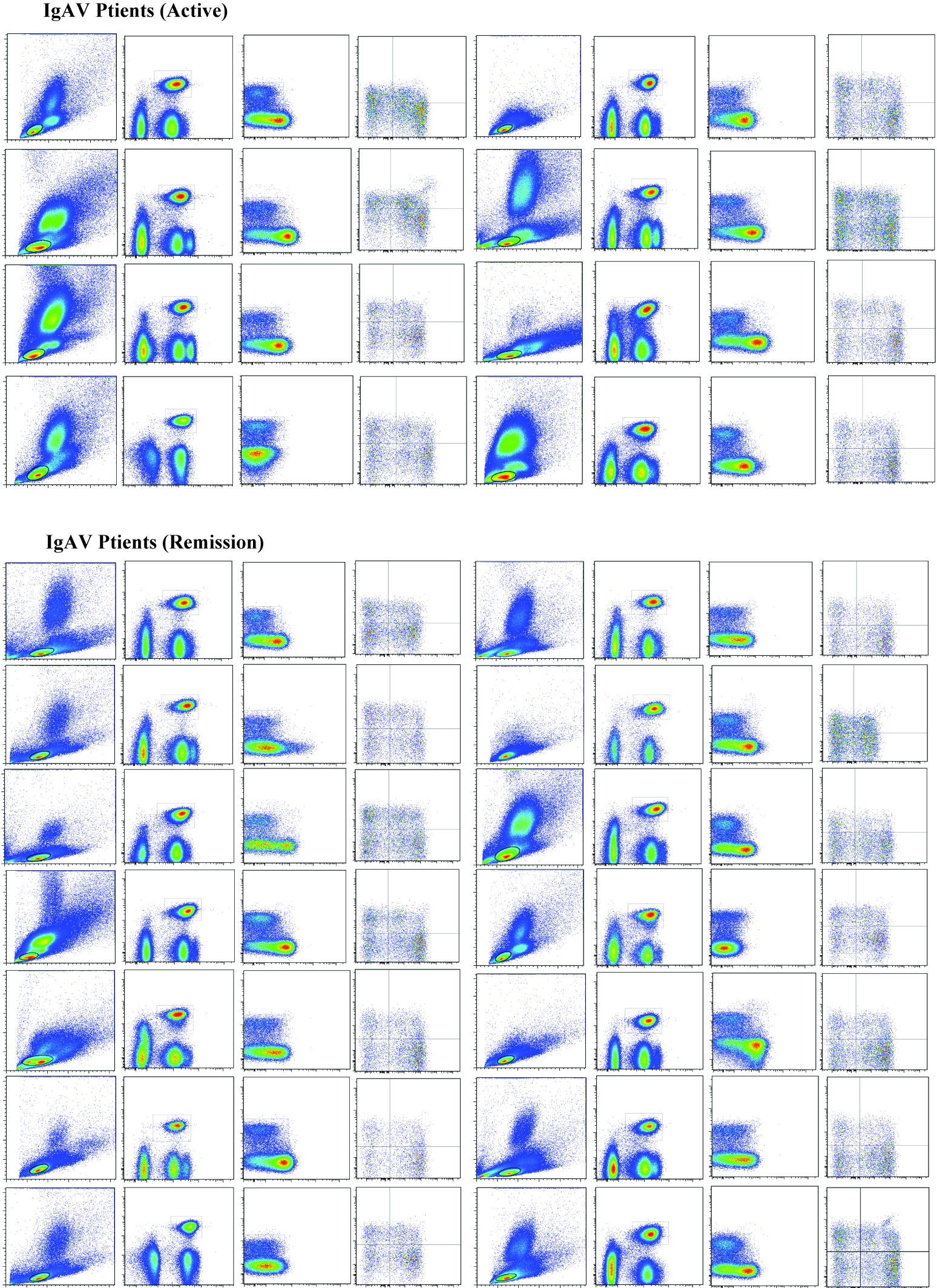

Supplement: S2 Fig — (TIF) [file pone.0189133.s002.tif]

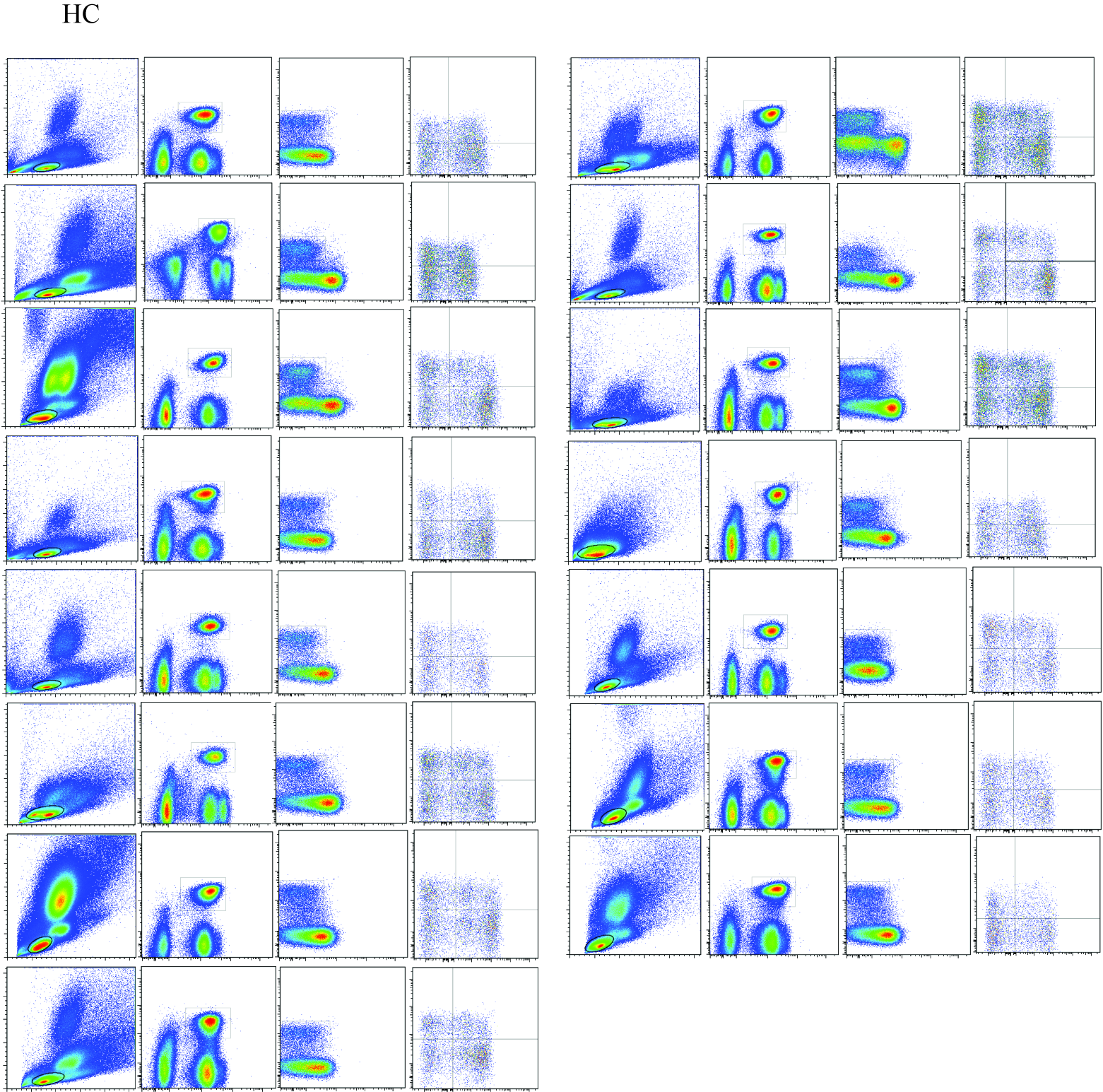

Supplement: S3 Fig — (TIF) [file pone.0189133.s003.tif]

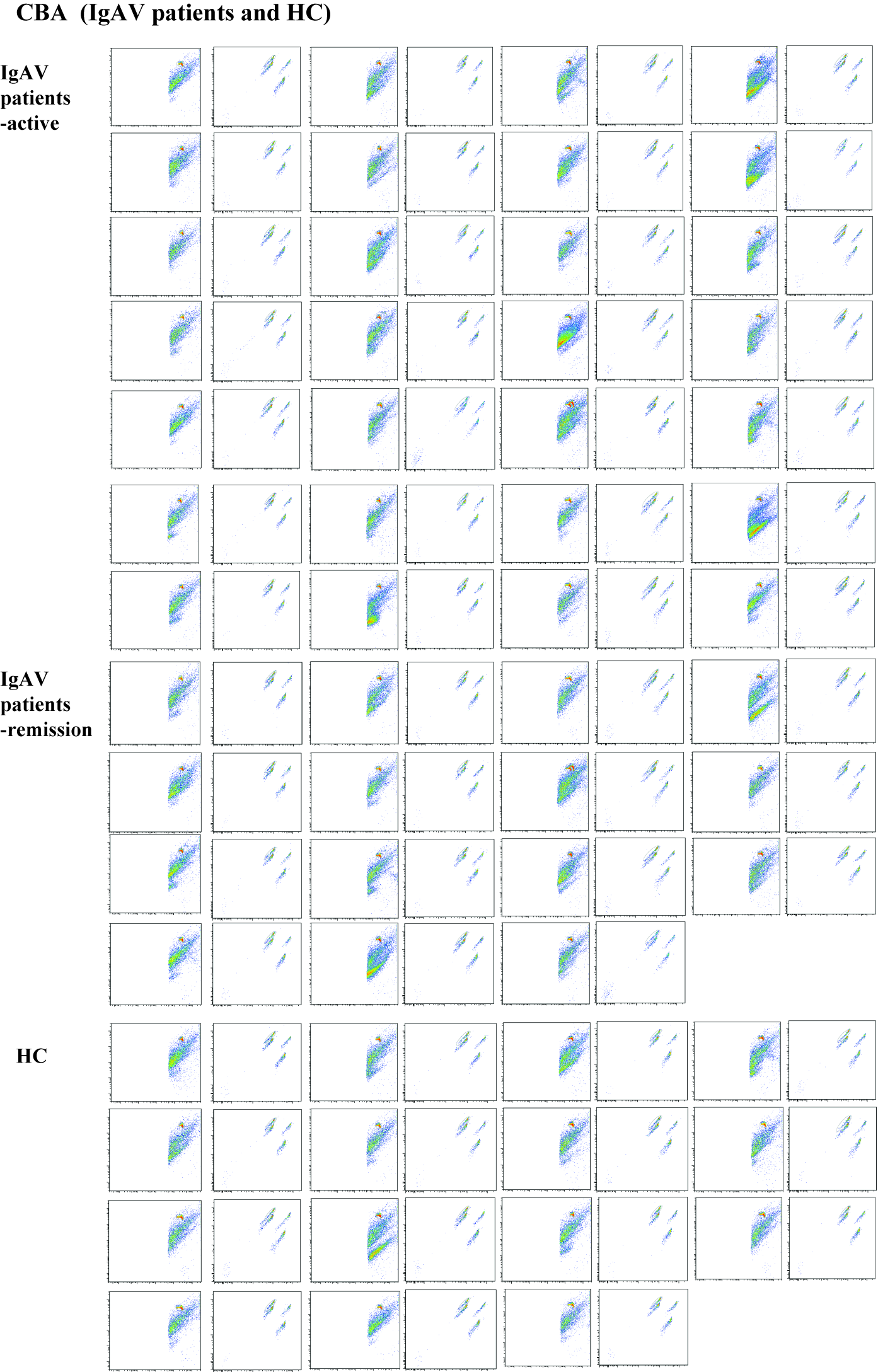

Supplement: S4 Fig — (TIF) [file pone.0189133.s004.tif]
